# 

**Published:** 1898-05

**Authors:** 


### Southern Medical Record

A Monthly Journal of Medicine and Surgery.

VOL. XXVIII. ATLANTA, GA., MAY, 1898. No. 5

BERNARD WOLFF, M. D., EDITOR.
ASSOCIATE EDITORS:
A. W. GRIGGS, M. D.
J. McFADDEN GASTON, M. D.
WILLIS F. WESTMORELAND, M. D
B. M. ZETTLER, BUSINESS MANAGER.

